# Association between bone mineral metabolism and vascular calcification in end-stage renal disease

**DOI:** 10.1186/s12882-021-02652-z

**Published:** 2022-01-03

**Authors:** Louise Aaltonen, Niina Koivuviita, Marko Seppänen, Heikki Kröger, Xiaoyu Tong, Eliisa Löyttyniemi, Kaj Metsärinne

**Affiliations:** 1 Kidney Center, Department of Medicine, Turku University Hospital, PL 52, Kiinamyllynkatu 4-8, 20521 Turku, Finland; 2 Turku PET Centre, University of Turku, Kiinamyllynkatu 4-8, 20521 Turku, Finland; 3 Department of Clinical Physiology, Nuclear Medicine, Turku University Hospital, PL 52, Kiinamyllynkatu 4-8, 20521 Turku, Finland; 4 Kuopio Musculoskeletal Research Unit (KMRU) Institute of Clinical Medicine, University of Eastern Finland, POB 1627, Kuopio, Finland; 5 Kuopio University Hospital, Kuopio, Finland; 6 Department of Biostatistics, University of Turku, Kiinamyllynkatu 10, 20014 Turku, Finland

**Keywords:** Aortic calcification score, Coronary artery calcification score, Dialysis, Bone histomorphometry, ^18^F-NaF PET

## Abstract

**Background:**

Development of vascular calcification is accelerated in patients with end-stage renal disease. In addition to traditional risk factors of cardiovascular disease (CVD) abnormal bone and mineral metabolism together with many other factors contribute to the excess cardiovascular burden in patients on dialysis. Aortic calcification score and coronary calcification score are predictive of CVD and mortality. The aim of this study was to evaluate the possible relationship between arterial calcification and bone metabolism.

**Methods:**

Thirty two patients on dialysis were included. All patients underwent a bone biopsy to assess bone histomorphometry and a ^18^F-NaF PET scan. Fluoride activity was measured in the lumbar spine (L1 – L4) and at the anterior iliac crest. Arterial calcification scores were assessed by computerized tomography for quantification of coronary artery calcification score and lateral lumbar radiography for aortic calcification score.

**Results:**

This study group showed high prevalence of arterial calcification and 59% had verified CVD. Both CAC and AAC were significantly higher in patients with verified CVD. Only 22% had low turnover bone disease. There was a weak association between fluoride activity, which reflects bone turnover, measured in the lumbar spine, and CAC and between PTH and CAC. There was also a weak association between erosion surfaces and AAC. No significant association was found between calcification score and any other parameter measured.

**Conclusions:**

The results in this study highlight the complexity, when evaluating the link between bone remodeling and vascular calcification in patients with multiple comorbidities and extensive atherosclerosis. Several studies suggest an impact of bone turnover on development of arterial calcification and there is some evidence of reduced progression of vascular calcification with improvement in bone status. The present study indicates an association between vascular calcification and bone turnover, even though many parameters of bone turnover failed to show significance. In the presence of multiple other factors contributing to the development of calcification, the impact of bone remodeling might be diminished.

**Trial registration:**

The study is registered in ClinicalTrials.gov protocol registration and result system, ID is NCT02967042. Date of registration is 17/11/2016.

## Introduction

Patients with end-stage renal disease (ESRD) have increased risk of death due to high prevalence of cardiovascular disease (CVD) [1–3]. Development of atherosclerosis and vascular calcification is accelerated in this patient group [4, 5]. In addition to traditional risk factors of CVD [6], abnormal bone and mineral metabolism together with uremic toxins, oxidative stress and inflammation contribute to the excess cardiovascular burden in patients on dialysis [7, 8].

Aortic calcification (AAC) increases with age [9] and has been associated with an increased risk of cardiovascular outcomes [10, 11]. The presence of arterial calcification is predictive of CVD and mortality [12–16]. AAC is an independent predictor of obstructive coronary disease and correlates with coronary artery calcification (CAC) [15, 17, 18]. Up to 80 – 90% prevalence of vascular calcification in ESRD patients has been reported [19]. Coronary calcification is associated with CVD, myocardial infarction and all-cause mortality also in the CKD population, including patients on dialysis [20–22]. CAC assessed by non-contrast cardiac computed tomography (CT) is highly sensitive for detection of coronary artery disease [23, 24].

Impaired bone metabolism and vascular calcification are intricately interconnected in patients with chronic kidney disease. Lower bone formation rate is associated with coronary calcification in CKD patients not yet on dialysis [25]. A few studies have suggested a link between low bone activity and arterial calcification and aortic stiffening in ESRD patients [26, 27], although the specific mechanisms are not yet fully understood. Improvement of turnover in both high and low turnover bone disorders, is associated with lower CAC progression in hemodialysis patients [25]. An inverse association between arterial calcifications and bone mineral density (BMD), trabecular bone score and trabecular bone volume has also been documented in uremic patients [20, 25, 28]. Calcium overload is associated with both the presence and the progression of arterial calcification [29, 30].

Bone metabolism is commonly assessed by measuring biomarkers or histomorphometric parameters obtained by bone biopsy. ^18^F- Sodium Fluoride positron emission tomography (^18^F-NaF PET) is a *novel* imaging technique that allows assessment of regional bone turnover [31–33]. ^18^F-NaF PET is the preferred imaging technology when studying quantitative molecular imaging of bone [34]. We have previously shown that ^18^F-NaF PET correlates with both static and dynamic histomorphometric parameters and can distinguish between low and high turnover bone disease [35, 36].

The purpose of this study was to evaluate the possible relationship between aortic and coronary calcification and bone qualities such as bone mineral metabolism and bone density measured by ^18^F-fluoride PET, bone histomorphometry and DXA in patients on dialysis.

## Methods

The study was approved by the Ethics committee of the Hospital District of South Western Finland and was conducted in accordance with the Declaration of Helsinki as revised 1964. The study is registered in ClinicalTrials.gov protocol registration and result system, ID is NCT02967042 and date of registration is 22/09/2016. All subjects gave written informed consent.

### Study subjects

The study subjects were recruited from the Kidney center in Turku. The inclusion criteria were: End-stage renal disease and dialysis treatment, biochemical abnormalities indicating mineral and bone disorder; long-term elevated PTH and hyperphosphatemia. Exclusion criteria were: pregnancy and bisphosphonate medication in the past 6 months. Ongoing medication for secondary hyperparathyroidism was continued, the medication remained unchanged during the study period. All patients underwent a ^18^F-NaF PET-CT scan including a CT scan of the heart. In addition, dual-energy X-ray absorptiometry (DXA) and a lateral lumbar x-ray were done. The bone biopsy was performed as a part of the study protocol within 4-6 weeks after the PET-scan.

### Laboratory assessment

Serum ionized calcium, alkaline phosphatase, phosphate, 25-hydroxyvitamin D, 1,25-dihydroxyvitamin D, intact parathyroid hormone, albumin, acid-base balance, full blood count, and creatinine were performed in all patients. The coagulation status was obtained previous to the bone biopsy. All tests were performed and analyzed by the local University Hospital laboratory. Biochemical markers were obtained in the morning or right before dialysis sessions.

### Abdominal aortic calcification score

Lateral lumbar radiography that included the abdominal aorta was performed using standard radiographic equipment. The AAC score was calculated using the scoring system described by Kauppila et al. [9]. In this method, the anterior and posterior aspects of the abdominal aorta are divided into four segments bound by the first four lumbar vertebrae. A total of 8 segments are evaluated (posterior and anterior wall). The degree of vascular calcification was graded 0, 1, 2 or 3, so that the total score can range from 0 - 24. In the anteroposterior severity score (0–24), the scores of individual aortic segments for the posterior and anterior walls were summed. All radiographs were analyzed by 2 independent researchers, and the mean AAC score was used in the analysis.

### Coronary tomography

All participants underwent a CT scan of the heart and coronary arteries prior to the ^18^F-NaF PET. CT scans were performed with a GE Discovery VCT 48 -slice CT/ positron emission tomography device (GE Healthcare). The coronary artery calcification score was calculated using the Agatston method for each coronary artery [37], and expressed in modified Agaston units (AU). Absence of CAC was defined as Agatston score of 0. Presence of CAC was defined as Agatson score of 1 or greater. CAC grade: 0, 1-100, 101 - 399, ≥400.

### Dual-energy X-ray absorptiometry (DXA)

DXA is an X-ray based imaging method, used widely for the diagnosis of osteoporosis [38]. BMD was measured with the use of dual X-ray absorptiometry (DXA) from the proximal femurs (femoral neck) and the lumbar spine (L1 – L4). DXA device (Hologic QDR 4500C, Hologic Inc., USA or Osteocore III, Medilink, France) was used. BMD was reported as grams per centimeter. Individual patient’s results were expressed as T-scores.

### Bone biopsy and bone histomorphometry

Iliac crest biopsies were performed vertically under local anesthesia including one cortex. The patients underwent fluorochrome double labeling by receiving 500 mg tetracycline three times daily for two days per os, followed by a drug free interval of ten days and a further two days administration of tetracycline. Bone biopsy was completed 7-10 days after the second label. The investigator double-checked before the procedure that tetracycline was taken as ordinated. Bone biopsies were obtained using a Snarecoil Mermaid Medical RBN-86 8G (3.3 mm) x15cm needle. Bone biopsies were fixed in 70% ethanol for at least 48 h before embedding in polymethylmethacrylate. The samples were cut into 5-μm thick sections and then stained with modified Masson-Goldner trichrome stain for static parameters, unstained sections were used for dynamic parameters. A semiautomatic image analyzer (Bioquant Osteo II, Bioquant Image Analysis Corporation, Nashville, TN, USA) was used for analyzing all parameters. In two patients, with only a single tetracycline label, we used a value for MAR of 0.3 μm/day in line with ASBMR Histomorphometry Nomenclature Committee recommendations for biopsies with only single labels [39]. The values for normal turnover were set using the results of Recker et co (mean ± 1SD) [40, 41]. The range for normal turnover in men was: BFR/BS 3.6 – 18.8 μm/y and Ac.f 0.12 – 0.6, in postmenopausal women: BFR/BS 6-22 μm/y and Ac.f 0.11 – 0.49 /y and in premenopausal women: BFR/BS 3-13 μm/y and Ac.f 0.04-0.26 /y.

### ^18^F-sodium fluoride positron emission tomography imaging

The PET scans were acquired using a Discovery VCT scanner (GE Healthcare). The tracer ^18^F-Fluoride ([^18^F]F^−^) is produced by 11-MeV proton irradiation of ^18^O-water using a cyclotron. The quality control tests for the ^18^F-NaF are conforming to the European Pharmacopeia. The subjects were positioned supine with the lumbar vertebrae in the field of view. A 60 min scan of the lumbar spine (L1-L4) followed by a 10 min static scan of the pelvis was done. The 60 min dynamic scan was begun simultaneously with an intravenous injection of 200 MBq ^18^F-NaF. The dynamic scan consisted of twenty-four 5-s, four 30-s and fourteen 240-s time frames. Low-dose CT-scans were done for image segmentation and attenuation correction. To generate bone activity curves (kilo becquerels per milliliter), regions of interest (ROI) in the lumbar spine were defined by drawing a ROI within each vertebral body, avoiding the end-plates and disk space. In the static PET scan of the pelvis ROI was defined by drawing a ROI on the anterior iliac crest, in the same region the bone biopsy was later obtained. We used an image derived input function by placing a ROI over the abdominal aorta (arterial input function, AIF) [42]. Patlak analysis was used to estimate the plasma clearance of ^18^F-Fluoride (net influx rate, K_i_) into the bone at the lumbar spine^38^. For the static scan of the pelvic bone; fractional uptake rate (FUR), which is an approximation of Patlak K_i_ [43], was calculated by dividing the bone activity concentration by area-under-curve of blood activity from ^18^F-Fluoride administration time to the time of static scan. Activity measurements were corrected for radioactive decay to the time of injection.

### Statistical analysis

Statistical analyses for background variables were performed using SAS 9.4 for Windows and JMP Pro 14. Characteristics of the study population were expressed as median and interquartile range (IQR)/range or mean and standard deviation (SD). For estimating the difference between means in different groups we used one-way analysis of variance (ANOVA).

Association between aortic and coronary calcification score, different bone qualities and CAC (and AAC) was studied with linear model including bone turnover variables (one at the time) adjusted with also sex and age. Assumptions for the model were checked using studentized residuals. Logarithmic transformation was used to some variables to fulfill the model assumptions. Pearson correlation coefficients were calculated between two continuous variables. In addition, bone turnover variables were compared between diabetic and non-diabetic patients were performed with two-sample t-test. The same method was used to compare low and high CAC (and AAC) values. P-values less than 0.05 (two-tailed) were considered as statistically significant.

## Results

### Patient characteristics

The study group comprised 32 end-stage renal disease patients. 50% were on peritoneal dialysis and 50% on hemodialysis. 41% were female and the average age was 66 years. 38% had diabetes and 22% were smokers. Patient demographics, kidney disease characteristics and medication are presented in Table 1.

**Table 1:** Demographics, disease characteristics and medication of the study group

No. of patients	.32
**Female sex** (%)	13 (41)
**Age, y** (median, range)	66 (37-83)
**BMI** (mean, SD)	24 (3.7)
**Smoker** (%)	7 (22)
**History of diabetes** (%)	12 (38)
**History of cardiovascular disease** (%)	19 (59)
**History of myocardial infarction** (%)	6 (19)
**Dialysis vintage,** month (median, IQR)	10 (6 - 30)
**Dialysis modality (PD/HD) (**%)	50/50
**Cause of ESRD**	
Diabetic nephropathy (%)	11 (34)
Hypertension/Arteriosclerosis (%)	4 (13)
Polycystic kidney disease (%)	7 (22)
Glomerulonephritis (%)	6 (19)
Other/unknown (%)	4 (13)
**Laboratory parameters**	
fS-calcium-ion 1.16 mmol/l - 1.30 mmol/l (mean, SD))	1.17 (0.08)
fP-phosphorus 0.71 – 1.23 mmol/l (median, IQR)	1.68 (0.48)
fP-iPTH 15 – 65 ng/l (median, IQR)	319 (188 – 508)
P- D-25 > 50 nmol/l (median, IQR)	70 (49 – 95)
S- D- 1,25 37 – 216 pmol/l (median, IQR)	31 (24 – 52)
P-tALP 35 – 105 U/l (median, IQR)	84 (57 – 128)
P-Albumine 36 – 45 g/l (median, IQR)	32 (28 – 34)
P-Magnesium 0.7 – 1 mmol/l (median, IQR)	0.9 (0.7 – 1.0)
cB-HCO3 22 – 26 mmol/l (median, IQR)	24 (22 – 26)
**Bone histomorphometry**	
High turnover/hyperparathyreoid bone disease (%)	11 (39)
Normal turnover/mild hyperparathyroid bone disease (%)	11 (39)
Low turnover/adynamic bone disease (%)	6 (22)
**Dynamic** ^**18**^**F-NaF PET**	
Lumbar spine, K_i mean_ L1-L4 (median, range)	0.040 (0.023 – 0.093)
Anterior iliac creast, FUR_mean_ hip (median, range)	0.041 (0.025 – 0.081)
**DXA BMD T-score**	
T-score L1-L4 (mean, SD)	−1.06 (1.58)
T-score femoral neck (mean, SD)	−2.0 (1.0)
**Osteoporosis** (T-score ≤ −2.5) (%)	10 (33)
**AAC score** (median, range)	8 (0 - 17)
**CAC score** (median, range)	397 (0 – 1980)
**Medication**	
Calcimimetic (%)	5 (16)
Alfacalcidol, Paricalcitol (%)	21 (66)
Calcium carbonate (%)	28 (88)
Cholecalciferol (%)	29 (91)
Sevelamer/lantane carbonate (%)	18 (56)
Corticosteroid (%)	2 (6)

Data is presented as mean (SD) for normal distribution variables or median (interquartile range) for non-normal distribution variables; *iPTH* intact parathyroid hormone, *tALP* total alkaline phosphatase, *DXA* dual energy x-ray absorptiometry, *BMD* bone mineral density

All patients were submitted to undergo a bone biopsy. In two patients the biopsy was unsuccessful and in two patients the biopsy was not sufficient to reliable analyze histomorphometric parameters. The histomorphometric characteristics of the study group are shown in Table 2. According to histomorphometric parameters, 39% had high turnover/hyperparathyroid bone disease and 22% had low turnover/adynamic bone disease. 13% had a mineralization defect.

**Table 2:** Histomorphometric characteristics (n = 28)

BFR/BS (μm^3^/μm^2^/year)	12.2 (0.95 – 39.3)
Ac.F/ yea	0.34 (0.1 – 0.83)
Ob.S/BS (%)	3.0 (0.0 – 17.4)
Oc.S/BS (%)	1.4 (0.0 – 6.7)
ES/BS (%)	2.9 (0 – 11.4)
MAR μ/day	0.8 (0.3 – 1.3)
MS/BS (%)	3. 6 (0.9 – 14.1)
Mlt (days)	40.1 (17.9 – 194.7)
OV/BV (%)	3.4 (0.5 – 10.6)
BV/TV(%)	19.6 (10.3 – 34.1)
O.th (Lm)	6.2 (3.7 – 11.0)
Tb.th (μm)	99.2 (56.1 – 172.1)

Values are expressed as median (range)

*BFR/BS* bone formation rate per bone surface, *Ac.f* activation frequency per year, *Ob.S/BS* osteoblast surface per bone surface, *Oc.S/BS* osteoclast surface per bone surface, *ES/BS* erosion surface per bone surface, *MAR* mineral apposition rate, *MS/BS* mineralized surface per bone surface, *Mlt* mineralization lag time, *OV/BV* osteoid volume of bone volume, *BV/TV* bone volume of tissue volume, *O.th* osteoid thickness, *Tb.Th* trabecular thickness

There was no statistically significant difference in histomorphometric markers obtained by bone biopsy, in turnover measured by ^18^F-NaF PET nor in T-scores measured in the lumbar spine and at the femoral neck when comparing patients with diabetes versus no diabetes and patient receiving hemodialysis versus peritoneal dialysis. tALP was significantly higher in patients with diabetes, the mean tALP in patients with DM was 146 (± 22) U/l and 84 (± 17) U/l in patients with no DM, p = 0.03. Pi was significantly higher in patients receiving hemodialysis treatment than in patients on peritoneal dialysis, Pi 1.8 (± 0.1) versus Pi 1.5 (± 0.1), p = 0.04.

### Vascular calcification and cardiovascular disease

**Fig. 1:**
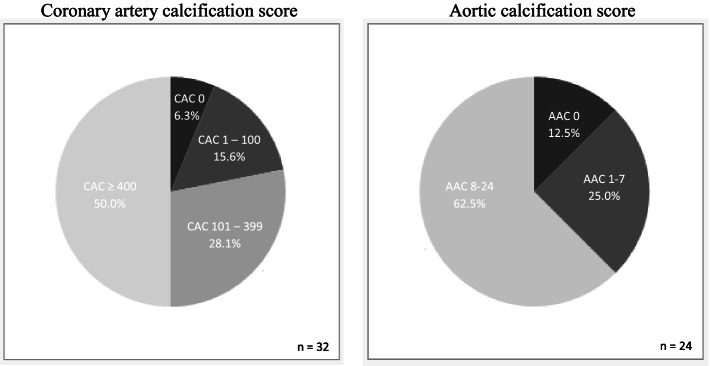
Distribution of coronary artery and aortic calcification scores. CAC = 0, CAC 1-100 = mild risk, CAC 101 – 399 = moderate risk, CAC ≥  400 = high risk. AAC = 0, AAC 1- 8 = moderate risk, AAC 8-24 = high risk

Coronary artery calcification (CAC) score was calculated in all patients. Only 6.3%, had CAC score = 0, 50% had CAC score ≥ 400, Fig. 1a. Aortic calcification score was estimated in 24/32 patients. 12.5% had AAC score = 0 and 62.5% had AAC score > 8/24, Fig. 1b. CAC and AAC correlated significantly, p < 000.1 and r_s_ = 0.63.

In this study population, 59% had a history of cardiovascular disease and 19% had a documented myocardial infarction. Both AAC and CAC score were significantly higher in patients with verified cardiovascular disease, p < 0.001 (CAC) and p = 0.03 (AAC). Also in the non-diabetic group, both AAC and CAC scores were significantly higher than in the group with verified cardiovascular disease, p < 0.001 (CAC) and p < 0.01 (AAC). DXA was obtained in 30/32 patients. 36% (5/14) of the patients with CAC ≥ 400 had osteoporosis. In patients with CAC 101 - 399 44% (4/9) had osteoporosis and in patients with CAC 1 - 100, 20% (1/5) had osteoporosis. 38% (5/13) of patients with AAC ≥ 8 had osteoporosis and 33% (2/6) of the patients with AAC 1 - 7.

We compared patients with high AAC score > 8 (n = 15) and patients with low AAC score (n = 9). We also compared patients with CAC > 400 (n = 16) to patients with CAC < 100 (n = 7), and patients with CAC > 100 (n = 25) to patients with CAC < 100 (n = 7). All histomorphometric markers (BFR, Ac.f, Ob.S/BS, Oc.S/BS, MS/BS, ES/BS, MAR, Mlt, OV/BV, O.Yh, Tb.Th, OV/BV), fluoride activity measured by ^18^F-NaF PET in the lumbar spine, K_i mean_ and the iliac crest FUR_mean,_ T-scores measured in the lumbar spine and at the femoral neck and laboratory parameters were analyzed. The only statistically significant finding vas, that BV/TV was slightly higher in patients with high AAC score, but no difference in patients with high or low CAC score.

### Association between AAC and CAC and bone metabolism and density measured by ^18^F-fluoride PET, bone histomorphometry, DXA and laboratory parameters

In the non-diabetic group, there was a correlation between CAC and age (p = 0.02, r_s_ = 0.52), indications of correlation between AAC and age was also observed, but did not reach statistical significance (p = 0.07. r_s_ = 0.51). Albumin correlated negatively with AAC (p < 0.01, r_s_ = −0.57), but not with CAC (p = 0.17, r_s_ = −0.25). No correlation could be shown between calcification scores and dialysis vintage, BMI and CRP.

Associations between aortic and coronary calcification score and different bone qualities were tested direct and with a model adjusted to age and sex. Tested bone qualities were turnover measured by ^18^F-NaF PET, histomorphometric markers (bone formation rate, BFR/BS, activation frequency, Ac.f, osteoclast and osteoblast activity, Ob.S/BS, Oc.S/BS, erosion surface, ES/BS, mineral apposition rate, MAR, mineralized surface, MS/BS, osteoid volume of bone volume. OV/BV), bone volume of tissue volume (BV/TV), osteoid thickness (O.th), mineralization lag time (Mlt) and trabecular thickness (Tb.th)), bone density (T-score at the lumbar spine and the femoral neck) and laboratory parameters. Calcifications scores and log-transformed calcifications scores were tested. There was a weak positive, but significant association between CAC and Ki, adjusted with age and sex, p = 0.01. There was also a weak positive association between CAC and PTH, p = 0.04 and between ES/BS and AAC, p = 0.04, when adjusted with age and sex.

## Discussion

The link between impaired bone metabolism and arterial calcification in patients with chronic kidney disease is well acknowledged, but still a matter of controversy. A recent study showed that the presence of vascular calcification affects several pathways in bone and is associated with lower bone density and impaired bone metabolism [44]. Several studies have suggested a link between bone turnover and vascular calcification in patients on hemodialysis, especially low turnover bone disease [26, 27, 45], but also contradictory results have been published [46–48]. In this study, there was a weak association between fluoride activity measured in the lumbar spine by ^18^F-NaF PET and CAC. There was also a weak association between erosion surfaces and AAC and between PTH and CAC.

Our cohort is a valid example of the current dialysis patients in Western countries. The patients in this cohort were rather elderly, median age 66 years and more than one third had a history of diabetes. More than 50% of the study population had manifested cardiovascular disease. The prevalence of coronary artery and aortic calcification was high and arterial calcification scores, both CAC and AAC, were significantly higher in patients with cardiovascular disease. 50% had CAC > 400, which suggest severe and extensive atherosclerotic disease. A half of the patients were on peritoneal dialysis and this differs from previous studies. This was a cross-sectional study. Both the bone biopsy and ^18^F-NaF PET was part of the study protocol and the range of PTH was narrow, with only a few very low or high values. According to histomorphometric markers obtained by bone biopsy, only 22% had low turnover/adynamic bone disease, which diverge from other studies indicating an association between arterial calcification and bone metabolism.

In the studies reporting a link between turnover and calcification score, London et co [26, 27] found that high arterial calcification score was associated with bone histomorphometry suggestive of adynamic bone disease. In this study, 43% of the patients had previously underwent parathyroidectomy. Furthermore, calcification score was evaluated ultrasonographically, which has relatively low sensitivity. They also reported that adynamic bone disease was associated with greater aortic stiffening and that aortic calcification scores were higher in this group. In the latter cohort, the study population was significantly younger than in our cohort. In addition, 50% had adynamic bone disease. Noteworthy is, that the subgroups of renal osteodystrophy were classified based on only tetracycline labels. In both cohorts, the study population was larger than in our study and the patients were nondiabetic and on hemodialysis treatment. In the study of Asci et co [45], over 200 patients on hemodialysis underwent a bone biopsy and CAC score was assessed. They found a significant positive correlation between histomorphometric bone turnover markers (BFR and Ac.f) and CAC in the whole study population. When analyzing only patients with CAC score > 0, there was a negative association between CAC and bone turnover markers (Ac.f and BFR) in low turnover patients, which was not found in patients with normal turnover. In addition, a positive correlation in high turnover patients was observed. Noteworthy is, that as much as 71% of the patients had low turnover bone disease, 27% had manifested cardiovascular disease compared to 50% in our study group and less than a third were diabetic.

In the studies published by Barreto and Coen [47, 48] there was no association between bone turnover parameters and CAC score. In the study of Barreto et al., the study population was rather young with a high prevalence of coronary calcification and 31% had a CAC score > 400. They found that higher osteoprotegerin was associated with coronary artery calcification, but no association with histomorphometric parameters of bone turnover. The cohort of the study of Coen was more similar to the present study population including 32 patients with a mean age of 55 years. Only 9% had low turnover bone disease. After multiregression analysis, no association between CAC score and bone turnover was observed.

In the present study, there was a weak association between fluoride uptake in the lumbar spine and CAC. Quantitative dynamic ^18^F-NaF PET measures bone turnover at multiple sites and gives a more extensive evaluation of the skeleton. However, there was no association between CAC and fluoride uptake at the iliac crest and no association between CAC and any of the histomorphometric parameters measured. There was a weak association between PTH and CAC. PTH is a biomarker of turnover, but it has limited ability to correctly estimate bone turnover except for the extreme high or low PTH levels [49]. The range of PTH in this study population was narrow and the significance of this result remain unclear. Overall, one should be cautious when interpreting the results keeping in mind that this was a cross-sectional rather small study. Still these results strengthen the assumption, that there is a link between vascular calcification and bone metabolism.

A strength of the present study is that bone turnover was measured with both bone histomorphometry and ^18^F-NaF PET imaging. Also the fact, that both coronary artery and aortic calcification were measured, is a strength. Both imaging methods are established and precise to detect arterial calcification. When it comes to the comparability of the studies evaluating bone metabolism and arterial calcification, one problem is the variability of the methods used to define calcification score and the variability in the classification of renal osteodystrophy.

A few studies have also reported a link between lower bone mineral density and vascular calcification in patients with CKD [19, 28, 50]. In this study, we did not find an association between bone density and vascular calcification. High prevalence of high risk arterial calcification, the heterogeneity of the study populations and the rather high median age, might diminish the association between bone density and vascular calcification.

The limitations of this study is the cross-sectional nature of the study, the relatively low number of patients and the heterogeneity of the study group. Also the small number of patients with low turnover bone disease and the fact, that only PTH was measured as a biomarker of turnover, are limitations.

## Conclusions

In summary, one can state that the association between arterial calcification and bone turnover still is controversial. The results of this study strengthen the assumption of a link between bone remodeling and arterial calcification, but also highlight the complexity, when evaluating patients with multiple comorbidities and extensive atherosclerosis. Also the fact, that only 22% had low turnover/adynamic bone disease in this study, might impact the significance of the results. Evidence of improvement in turnover and reduced progression of vascular calcification^25^ and the association between low turnover and vascular calcification^45^ strengthen the assumption of a link between vascular calcification and bone remodeling also in end-stage renal disease patients. However, in the presence of multiple other factors contributing to the development of calcification, the impact of bone remodeling might be diminished. Prospective studies are needed to verify the impact of changes in bone turnover on the progression of vascular calcification.

## Data Availability

The data that support the findings of this study are available from the corresponding author upon reasonable request.

## Abbreviations

CVD: Cardiovascular disease
AAC: Aortic calcification score
CAC: Coronary calcification score
^18^F-NaF PET: ^18^F-Sodium Fluoride Positron Emission Tomography imaging
CT: Computerized tomography
DXA: Dual-energy X-ray absorptiometry
BMD: Bone mineral density
ESRD: End-stage renal disease
CKD: Chronic kidney disease
PTH: Parathyroid hormone
BFR/BS: Bone formation rate per bone surface
Ac.f: Activation frequency
Ob.S/BS: Osteoblast surface per bone surface
Oc.S/BS: Osteoclast surface per bone surface
ES/BS: Erosion surface per bone surface
MAR: Mineral apposition rate
MS/BS: Mineralized surface per bone surface
OV/BV: Osteoid volume per bone volume
BV/TV: Bone volume per tissue volume
O.th: Osteoid thickness
Tb.th: Trabecular thickness
